# Assessment of Donkey *(Equus asinus)* Welfare at Slaughter in Ghana

**DOI:** 10.3390/ani14243673

**Published:** 2024-12-19

**Authors:** Katharine Fletcher, Georgina Limon, Eric Agongo, Anthony Akunzule, Gloria Essel, Barbara Padalino, Andrew Grist, Troy John Gibson

**Affiliations:** 1 Animal Welfare Science and Ethics Group, Department of Pathobiology and Population Sciences, Royal Veterinary College, Hawkshead Lane, Hatfield AL9 7TA, UK; tgibson@rvc.ac.uk; 2 Veterinary Epidemiology, Economics and Public Health Group, Department of Pathobiology and Population Sciences, Royal Veterinary College, Hawkshead Lane, Hatfield AL9 7TA, UK; glimon@rvc.ac.uk; 3 The Pirbright Institute, Woking GU24 0NF, UK; 4 Ghana Poultry Network (GAPNET)/Food and Agriculture Organisation (FAO), Accra P.O. Box CT 5505, Ghana; ericagongo77@gmail.com (E.A.); anthony.akunzule@fao.org (A.A.); ntifua85@gmail.com (G.E.); 5 Department of Agricultural and Food Sciences, University of Bologna, Viale Giuseppe Fanin 46, 40127 Bologna, Italy; barbara.padalino@unibo.it; 6 Faculty of Science and Engineering, Southern Cross University, Lismore, NSW 2480, Australia; 7 Animal Welfare and Behaviour Group, School of Veterinary Sciences, University of Bristol, Langford BS40 5DU, UK; andy.grist@bristol.ac.uk

**Keywords:** equine welfare, *Equus asinus*, slaughter, Ghana, post-mortem, veterinary pathology, blunt force trauma

## Abstract

Donkey slaughter in West Africa has received limited scientific attention, despite increasing over recent years. This study aimed to explore factors affecting donkey welfare, both ante-mortem and at slaughter, in the Upper East region of Ghana. A total of 134 donkeys at five different slaughter points were assessed using animal-based indicators. Slaughter involved either blunt force trauma (BFT) using a wooden pole (1 location) or a metal hammer (2 locations); or non-stun ventral neck incision (VNI) (2 locations). Time to loss of consciousness (or return) was assessed, with behavioural and brainstem signs of sensibility/consciousness. Negative human–animal interactions occurred ante-mortem, with animals struck multiple times and handled using aversive methods. Donkeys hit on the head with the wooden pole were five times more likely to be ineffectively concussed (*p* < 0.001, OR: 5.4, CI: 1.9–15.4) compared to the hammer and took significantly longer to lose consciousness (*p* < 0.001). The mean time to loss of corneal reflex for those hit by wooden pole was 166.9 s (SD 21.1; range: 79–425 s), compared to just one animal displaying corneal reflex for 59 s after being hit by metal hammer. For those animals slaughtered through VNI, corneal reflex was observed for a mean time of 96.5 s (SD 4.3, range 26–164 s). The findings of this study highlight major concerns regarding the methods used for donkey slaughter in the Upper East region in Ghana, particularly regarding aversive ante-mortem handling and prolonged time to loss of consciousness during VNI and ineffective BFT in inducing unconsciousness by concussion.

## Simple Summary

Donkey slaughter in Ghana was assessed through observing human–animal interactions prior to slaughter, and different methods of slaughter (slaughter without pre-stunning and two different methods of blunt force trauma to the head) were evaluated. Animals were hit multiple times by personnel during handling and movement. At slaughter, animals killed through neck cutting without pre-stunning showed signs of consciousness up to a mean time of 96.5 s. Animals experiencing blunt force trauma to the head took up to 166.9 s to lose consciousness when using a wooden pole. This method was five times more likely to be ineffective compared to blunt force trauma with a metal hammer. This study shows that donkeys in Ghana currently experience welfare compromise during all stages of the slaughter process; in particular, through aversive handling practises and ineffective slaughter methods, which delay the time to loss of consciousness and potentially increase suffering.

## 1. Introduction

Donkeys (*Equus asinus*) are routinely slaughtered in many countries, for both their meat and hide for the preparation of eijao, a donkey-hide gelatin used in traditional Chinese medicine. The Food and Agricultural Organization (FAO) estimates that 981,995 donkeys were slaughtered in China in 2021, with Niger slaughtering 149,532, Mali slaughtering 61,470, and data not existing for some other African countries, including Ghana [1]. However, these are just approximate figures, with donkey slaughter largely unregulated and often unrecorded, and the demand for eijao resulted in an estimated five million donkeys slaughtered globally each year [2].

Many donkeys are sourced from Africa, which traditionally has two-thirds of the world’s donkey population [3] with their skins exported to China, as domestic supply is insufficient to meet the demand for eijao [4]. Some African countries have banned the slaughter of donkeys for their skins; however, with porous borders and a lack of enforcement, trade continues [3]. Ghana officially prohibits the slaughter of donkeys specifically for their skins, but donkey meat is still widely consumed, and it is still considered one of the key countries involved in the donkey skin trade [5,6].

Previous studies have found that people involved in animal slaughter in Africa have very limited knowledge of animal welfare [7] and receive no training on either animal welfare or slaughter practises [8]. Resources are also limited [9], with African abattoirs often lacking access to purpose-built stunners such as Captive Bolt Guns (CBG). Stunning is, therefore, often attempted through blunt force trauma (BFT), defined as the impact of an object with a blunt surface against a head [10]. The level of damage this can cause is dependent upon the mass and velocity of the object and whether the animal is stationery or moving. Ventral neck incision is then conducted as a secondary method. However, some studies have questioned the effectiveness and humaneness of BFT [11] amidst concern that animals can regain consciousness through the slaughter process [12], particularly where there are delays between BFT and bleeding.

Many animals in Africa are bled without pre-stunning [8,13], through ventral neck incision (VNI), which causes haemorrhage-induced cerebral hypoxia, slowing and then disrupting brain activity and producing irrecoverable unconsciousness [14]. However, non-stun slaughter through VNI is known to cause pain and stress, even where vessel severance is conducted quickly and cleanly [15,16,17].

For slaughter to be considered humane, an animal must not regain consciousness until death [16]. However, the effectiveness or humaneness of VNI or BFT has not been systematically assessed in donkeys, nor have other slaughter practices and conditions for donkeys in Africa, with a recent systematic review finding no published studies on donkey slaughter and/or or in low-middle income countries [18]. Moreover, current regulations on the welfare of equids are very limited are not equid-specific, and the World Organisation for Animal Health (WOAH)’s present guidance on the slaughter of animals does not include donkeys [19]. Research into this area is, therefore, urgently required to inform future policy and legislation and to help improve the welfare of equids at slaughter across the world.

### Aims

This is an exploratory study with the overall aim of conducting animal welfare assessment at donkey slaughter points, both at ante-mortem and at slaughter, in the Upper East region of Ghana. The specific aims were:

- To assess human–animal interactions, handling methods, and stress-related behaviours displayed by donkeys ante-mortem/prior to slaughter and to determine if there is an association between these factors.
- To evaluate signs of consciousness/brainstem activity displayed in donkeys following BFT and/or VNI and to determine variations between two different BFT methods; (i) wooden pole, and (ii) metal hammer
- To determine the time to loss of consciousness for donkeys slaughtered through VNI and the effectiveness of this method.

## 2. Materials and Methods

### 2.1. Ethical Statement

Ethical approval for this study was granted by the Royal Veterinary College, Clinical Research Ethical Review Board (URN 2022 2103-3) and Social Science Research Ethical Review Board (URN: SR2022-0059). Consent was obtained from the person in charge of each slaughter point, prior to data collection, and from each interviewee prior to conducting structured questionnaires.

### 2.2. Sample

Sample size was calculated to determine differences on stunning effectiveness between two BFT methods. Assuming 30% difference between the two groups, for example, 10% in group 1 (metal hammer) and 40% in group 2 (wooden pole), having 29 animals in each group allows us to identify differences with 95% confidence and 80% power. For the VNI technique, we aimed to collect data in at least half of the animals slaughtered using this technique on each day of data collection at that same location in order to achieve an estimation on the time to loss of consciousness.

### 2.3. Data Collection

Data were systematically collected from donkey slaughter points, and between 50 and 210 animals were processed per week. Information regarding the origin of each animal was not available. The ante-mortem protocol was piloted and refined on the first day, so only two animals from this day were included for this stage.

### 2.4. Holding Pen/Lairage

Lairage holding pen dimensions and stocking densities were recorded. Temperature and humidity were measured using a thermometer and humidity reader (Kestrel 4000, Kestrel Instruments, Nielsen-Kellerman, Boothwyn, PA, USA). The presence of food, water, bedding, and shelter were recorded for each lairage area as present/absent at the start of each day of data collection. Cleanliness of the pen was subjectively scored as poor (multiple faeces and severe hazards), moderate (minor hazards and some faeces), or good (minimal faeces and minimal/no hazards) [20]. Lighting was assessed using a Luxmeter (Delta OHM, Luxmeter HD 8366, Senseca, Caselle di Selvazzano, Italy).

Donkey health was assessed ante-mortem by observations at a distance, with animal-based indicators of health issues—ocular discharge, nasal discharge, abnormal respiration, signs of disease/infection, skin/coat issues, lesions/wounds, scars/swellings, foot/limb abnormalities and signs of lameness—recorded as present/absent, along with Body Condition Score on a 5-point scale from 1 (poor) to 5 (obese) [21], based only on visual inspection.

### 2.5. Pre-Slaughter Behaviour/Restraint

The welfare assessment protocol used was developed following a systematic review of the literature [18], which drew on published equine welfare assessments and other literature. Animal-based indicators were combined with welfare inputs to assess factors that would be difficult to assess through animal observations alone. The protocol was first tested in a field setting, for horses at an abattoir in the United Kingdom, with further refinement at an abattoir in Italy. However, adaptations were made to allow for frequent behaviours observed in donkeys and to accommodate parameters relevant to the slaughter methods used in Ghana, with the accessibility and practicability of assessing each individual parameter tested under these conditions and adapted accordingly.

Conspicuous behaviour sampling was used, with presence/absence recording conducted for the total time the animal was observed to be handled until the time they were either stunned using BFT or killed using VNI (Table 1). The method of handling (including details in how it was conducted) was recorded. Individual donkeys were not followed through each phase of the process. The research team consisted of two assessors with extensive behavioural and welfare assessment experience. Each team member was responsible for collecting data at one area throughout the study. All observations were recorded using a combination of recording sheets completed by hand and a dictaphone (Olympus VN-713PC, Olympus, Hachioji-shi, Tokyo, Japan) and headset (Sennheiser PC2, Sennheiser electronic GmbH & Co, Wedemark, Germany). Data were then entered onto an Excel spreadsheet by the primary researcher.

The human–animal interactions and material used were assessed using the indicators presented in Table 2 for the duration of time in which the animal was either moved to the slaughter area or prior to/during slaughter until such time as blunt force trauma or slaughter without stunning was performed. Animals were restrained as per usual practice, with the method of restraint recorded accordingly. Assessors stood at a distance of ≥1 m so as not to interfere with routine practice at the slaughter point.

### 2.6. Slaughter

The presence or absence of BFT along with the type, length, and diameter of any equipment used to do so, the number of attempts to stun, and the animal’s response to human handling were recorded. The mass/weight of the BFT device was not measured. The type, length, and condition of the knife were recorded (good = clean, sufficiently sharp, no jagged edges; moderate = relatively clean/sharp but well used; poor = unclean, blunt or jagged) with any nicks on the knife counted. The number of cuts (movement of the knife in one direction) was also recorded and so was the cut position relative to the trachea rings. The trachea rings were counted from the arytenoid cartilage onwards (position 0, followed by 1 as from the first trachea ring) but not including the cricoid cartilage. The right and left carotid arteries, jugular veins, trachea, oesophagus, and spinal cord were assessed and recorded as intact (including where partially intact), stretched/disarticulation (for spinal cord), or severed. The presence of a false aneurysm (carotid occlusion) was also recorded.

The time from restraint to VNI or from BFT to VNI was recorded along with the time from the first hit and/or first cut to cessation of reflexes, using a digital stopwatch (Guang Cai Lun ZSD-809, Jeanoko, Longgang, Shenzhen, China). Immediately after stunning and/or VNI, the animals were continually assessed sequentially (every 5 s) for signs of sensibility/insensibility as detailed in Table 3 until complete cessation of responses.

Where BFT had occurred, the heads were removed as part of routine processing, and then a subset were examined in situ at the slaughter point, with heads not able to be traced back to the same animals assessed behaviourally at slaughter. Deviation from the Humane Slaughter Association (HSA)’s suggested shooting/stunning position for horses was recorded as there are no published equivalent guides for donkeys. This is stated as 20mm above the intersection of lines drawn from the middle of each eye to the base of the opposite ear [34]. The position of the hit was first determined by marking the recommended stunning position on the head, along with the actual point of hit, with the difference between them measured. Photographs of the heads were taken on a digital camera (Olympus IM015 TG-6, Olympus, Hachioji-shi, Tokyo, Japan). It was not possible to examine the brains for gross macroscopic or microscopic damage due to the heads being retained by the slaughter plants for commercial sale or private consumption.

### 2.7. Structured Questionnaires with Slaughter Personnel

Slaughter personnel (including the owner/manager of the slaughter point, the operator/technician responsible for each stage, e.g., BFT and cutting/bleeding) were asked to consent to participation in a face-to-face interview using a structured questionnaire. The questionnaire consisted of a combination of quantitative (closed-ended) and qualitative (open-ended) questions and gathered information on current practises and management, as well as about the individuals’ attitudes and behaviour regarding donkey slaughter and operator safety (see Supplementary Materials). The questionnaire was translated into the local language, Gurune (frafra), by a native Ghanaian Gurane (frafra) speaking member of the research team. Who asked the questions and translated answers back to English, which were recorded by the first author.

### 2.8. Data Handling and Statistical Analysis

Data were entered onto a Microsoft^®^ Excel^®^ (Version 2208) spreadsheet by the first author. To assess human–animal interactions and different handling methods ante-mortem, descriptive statistics were conducted. All continuous data were tested for distribution, with non-normally distributed parameters summarised through median and interquartile range and normally distributed parameters summarised through mean ± Standard Deviation or Standard Error, where appropriate.

To determine if there was an association between stress behaviours displayed by donkeys and ante-mortem factors such as environment or handling/human–animal interactions, Chi-squared (or Fisher’s Exact as appropriate) tests were performed. A modified one-way Anova for binary outcomes with Tukey post hoc tests were performed to determine if there was a difference between frequency of behavioural variables across locations (A–E). Kendall’s tau-b was used to assess the level of correlation between ante-mortem behavioural variables, with significant correlations determined to be where *p* ≥ 0.05.

To evaluate signs of consciousness/brainstem activity displayed in donkeys following BFT and/or VNI and to determine the time to loss of consciousness, descriptive statistics were conducted, and the distribution of data was tested through frequency histograms. Mann–Whitney (independent sample) tests were conducted to measure differences between locations (and/or type of BFT) for (i) number of BFT attempts; (ii) time from hit to cut; (iii) time to loss of consciousness. Linear regression was conducted to explore associations between (i) time taken from first impact to first cut and animals who showed signs of a return to consciousness, (ii) presence of false aneurysms and position of neck cut. Chi-squared (or Fisher’s Exact as appropriate) tests were performed to (i) determine any associations between VNI positioning and proportion of false aneurysms or occlusion, (ii) determine any associations between different methods of BFT (metal claw hammer or wooden pole) and signs of consciousness. As locations A and B used ‘non-stun’ exclusively, location C used ‘wooden BFT’ method exclusively and locations D and E use metal BFT exclusively, slaughter points were categorised into three different groups dependent upon method: ‘non-stun’, ‘wooden BFT’ and ‘metal BFT’ and were not adjusted by location. Animals were classified as ineffectively stunned after BFT if they failed to collapse and/or rhythmic breathing was present and/or if at least two of the following parameters were present: positive corneal reflex, positive palpebral reflex, eyeball rotation and nystagmus [35]. Kaplan–Meier survival curves were used to visualise the time to loss of consciousness signs according to BFT methods (metal hammer vs. wooden pole).

For the questionnaire analysis, each questionnaire was analysed individually with common answers grouped together and summarised. Quantitative answers, e.g., number of years in role, were expressed through mean ± Standard Deviation or Standard Error, where appropriate. SPSS (IBM SPSS Statistics 28.0.0.0, 2022) was used for all analysis. *p* ≤ 0.05 was used as the indicator of significance.

## 3. Results

A sample of 134 donkeys was assessed during routine slaughter at five slaughter points in the Upper East region of Ghana over a period of nine days in November 2022.

### 3.1. Ante-Mortem

The mean temperature over the course of data collection was 24.5 °C ± SD: 4.6 (range: 21.0–32.5 °C), with mean humidity at 42.8% SD: 9.1 (range: 35–58%. The mean lux was 5.5 SD: 11.9 (range: 0.0–37.9) Lx, with the earliest slaughter beginning at 03:00 hrs (AM) and the latest starting at 05:50 hrs.

**Figure 1:**
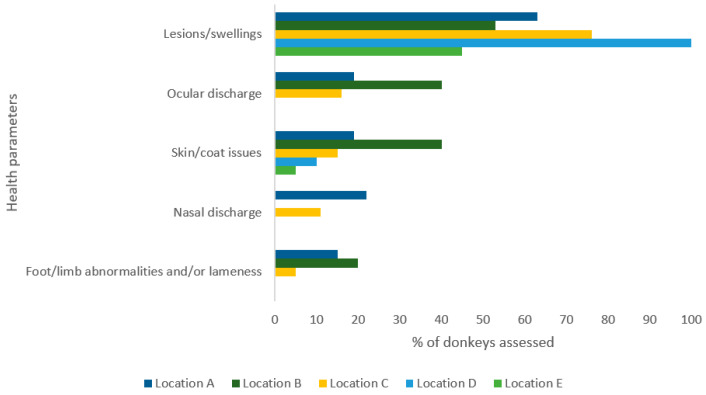
Health parameters logged for animals at each slaughter point location (N.B. ocular or nasal discharge or lameness not assessed at locations D and E due to visibility).

Stocking density was scored as adequate at all slaughter points during the entire sampling period (this was assessed based on the ability to turn fully in the pen or have room for one animal’s length and width between one another [20]). Vision/lighting was scored as poor on all but two occasions (2/9; 79%) at all locations except location A. Location A was scored as moderate once and good once, when slaughter started later in the morning, however this meant that higher temperature was simultaneously recorded at that time on those days. Cleanliness of pens/presence of faeces or hazards was scored as moderate in all but location C (89%; 8/9) where it was scored as good. Food, water, bedding, and shelter was not provided in the pens in each of the five locations. Sites D and E were not assessed for ocular/nasal discharge or foot abnormalities due to visibility/lighting restrictions. Thirty eight percent of animals had a BCS of ≤1.5, with no animals having a BCS of >2.5. A high proportion (total across sites: 66%) also showed lesions, scars, or swellings (Figure 1). In some cases, the lighting was too poor to accurately assess animals’ health parameters.

#### 3.1.1. Human–Animal Interactions

The most commonly observed behaviours prior to slaughter were rapid breathing (94%; 122/130), blinking (88%; 105/120), tail tuck (70%; 91/130), and ears flat back (65%; 84/130). A total of 8 animals (6%) (2 at location A, 5 at location D, and 1 at location E) were seen to slip, and 16 (12%) animals (6 at location A, 5 at location C, and 5 at location D) were seen to fall. All (100%; 134/134) animals reacted in a nervous/avoidant way towards the operator(s), attempting to move their head or body away. Although multiple different operators were conducting both handling and slaughtering, it was not possible to record the operators and measure the operator effect.

Environmental factors such as poor lighting/visibility complicated and, in some cases, particularly at locations A, D, and E, compromised recording reliability. Due to logistics and with large numbers of animals sometimes moved at the same time, it was not always possible to record all behavioural parameters for each individual animal and was considered missing data during the analysis.

Different methods were used to restrain animals prior to slaughter or move them from lairage to the slaughtering area. This included being hobbled for 28 (80%; 28/35) animals at location A and 29 (54%; 29/54) animals at location C. Animals were also pulled by a foreleg, ear, or tail. At location C, 30% (16/54) of animals were seen to be pushed/shoved, 39% (21/54) of animals were dragged along the ground, and 5% (3/54) of animals had their nasal planes pinched. Location E also had 90% (18/20) of animals dragged along the ground prior to slaughter. Restraint methods included leg tethering, rope casting, neck rope, kicking animals, chasing or herding, picking up the hind legs in a “wheelbarrow”, mounting or riding the animal, or use of other physical force.

A negative attitude (talking/shouting impatiently, forceful use of stick/hand) towards the animals was seen in 98% (132/135) of cases, with 73% of interactions (98/135) involving shouting at the animals (Table 4). Each animal was hit (to move) between 1 and 34 times, with a median of 7 (IQR: 9) (Table 5). The median was 6 for location A (IQR: 7.75, range: 1–24), 6.5 for location B (IQR: 9.5, range: 1–32), 6.5 for location C (IQR: 5.75, range: 1–34), 8.5 for location D (IQR: 7.5, range: 1–15), and 7 for location E (IQR: 9.5, range: 1–27). The total number of hits for each animal was not significant (*p* = 0.58).

#### 3.1.2. Associations Between Behaviours and Human–Animal Interactions

Behaviours were tested for correlation (Table 6). To address the aim of determining if there was an association between stress behaviours displayed by donkeys and location, Chi-squared tests were conducted, with significant associations (*p* > 0.05) found between all locations for all behaviours. Post hoc analysis was performed to look at differences between locations (Table 7).

### 3.2. Slaughter

Different types of knives and machetes were used for VNI, with three locations conducting BFT prior to VNI, using either a wooden pole or a metal claw hammer. In total, 68% (91/134) of animals were slaughtered in groups/alongside conspecifics, with 32% (43/134) isolated from conspecifics for slaughter.

#### 3.2.1. Non-Stunned Slaughter

Two locations used VNI, without prior stunning, as the slaughter method. This involved a hind leg being grasped, the animal tipped onto their side, and the ventral mandibular or larynx region being stood on prior to VNI. Knives were sharpened on other knives rather than purpose-built sharpening devices, and operators were not seen to clean the knife between animals. Knives at both location A and location B were assessed as being in a poor condition, with < 5 nicks, and the frequency of sharpening was every animal. Knives at location A had a blade length of 162–189 mm, with a 564 mm machete used at location B.

For those animals slaughtered by VNI, corneal reflex was observed for a mean time of 96.5 s (SD 4.3, range 26–164 s) after the first cut (Table 8). However, there was no significant difference in the time to loss of consciousness signs between locations A and B. No animals at location B showed nystagmus, blinking, or attempted rhythmic breathing. In total, 70% of animals lost jaw tone within 50 s of the first cut.

Two animals at location A did not have the left carotid artery severed from the cut. They continued to have positive palpebral (58 and 74 s) and corneal reflexes (98 and 119 s) after VNI. One showed a jaw spasm at 111 s post-cut. Rhythmic breathing was absent in both these animals. In total, 75% (27/36) of donkeys at location A had their spinal cord cut, whereas none of the donkeys at location B did. Location A cut higher up on the neck, above the trachea, at the back of the larynx into the arytenoid cartilage (Table 9). Only one animal at location A was observed as having a false aneurysm, with none at location B. There was a significant difference in the number of incisions per donkey between location A (6.8 ± (SE) 0.5, range 2–15) and location B (3.6 ± (SE) 0.3, range 3–5) (*p* < 0.001).

#### 3.2.2. Blunt Force Trauma Slaughter

Location C used a wooden pole with a length between 550 and 608 mm and a diameter of 28mm. Mass/weight was not measured. Location D used a standard metal claw hammer with a diameter of 25 mm, and location E used a welded/weighted metal claw hammer with a diameter of 33 mm. Locations D and E were combined for further analysis of BFT due to the same method being used at both locations (metal claw hammer) and a similar variation being observed.

The mean number of BFT attempts was 1.6 ± (SE 0.1, range 1–5) (Table 10), with 34% of animals (30/87) receiving more than one attempt. This was the most prevalent at location C, using the wooden pole, with 43% (25/58) of animals requiring multiple attempts, in comparison to 17% (5/29) at locations D or E, using the metal claw hammer (*p* = 0.003).

**Figure 2:**
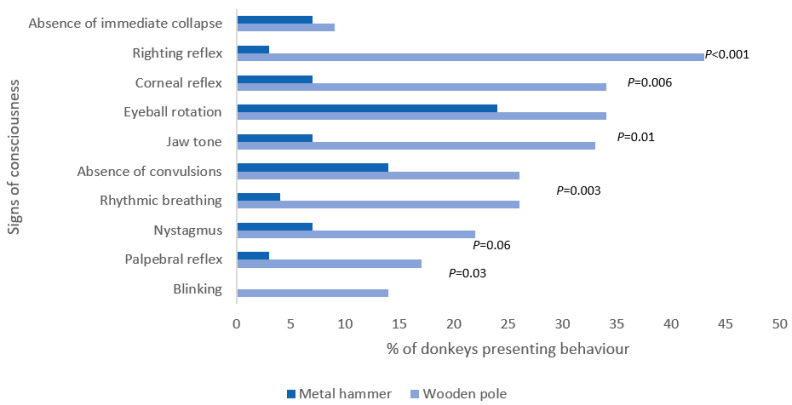
Signs of consciousness post-BFT (taken from first hit attempt; eye movements assessed from right-hand side) with *p*-value determined through the Chi-squared test to see if there were differences between locations for presence/absence of signs of consciousness.

After BFT with the wooden pole (location C), significantly more animals showed righting and corneal reflexes and attempted rhythmic breathing than animals experiencing BFT with a metal hammer (locations D and E) (Figure 2).

After BFT with the wooden pole, 41% (24/58) of animals righted themselves, compared to just one with the metal hammer. For animals hit with the wooden pole, 7% (4/58) showed a return of palpebral reflex, 28% (16/58) a return of corneal reflex, 19% (11/58) a return of blinking, and 21% (12/58) a return of rhythmic breathing, compared to one each (1/29), respectively, for those stunned with the metal hammer. Nystagmus was present post-impact for 17% (10/58) of those hit with the wooden pole, compared to 7% (2/29) with the metal hammer. Eyeball rotation returned for 34% (20/58) after the wooden pole compared to 14% (4/29) with the metal hammer, and jaw tone returned for 17% (10/58) of those with the wooden pole compared to 7% (2/29) for those after the metal hammer. More animals were classed as ineffectively stunned with the wooden pole compared to the metal claw hammer (*p* = 0.001, OR: 5.3, CI: 1.9–15.4).

The mean time to loss of corneal reflex for those hit by wooden pole was 166.9 s (SD 21.07; range: 79–425 s), and it took up to 273 s for rhythmic breathing to cease, compared to 59 s (*n* = 1) and 87 s (*n* = 1), respectively, for those hit by metal hammer. Animals hit by the wooden pole also showed a return to sensibility up to 254 s after the first hit (see Supplementary Materials).

For animals that showed a return of jaw tone, this was seen within 50 s for 50% of animals stunned with the wooden pole and for the two animals showing a return of this after being stunned with the metal hammer.

**Figure 3:**
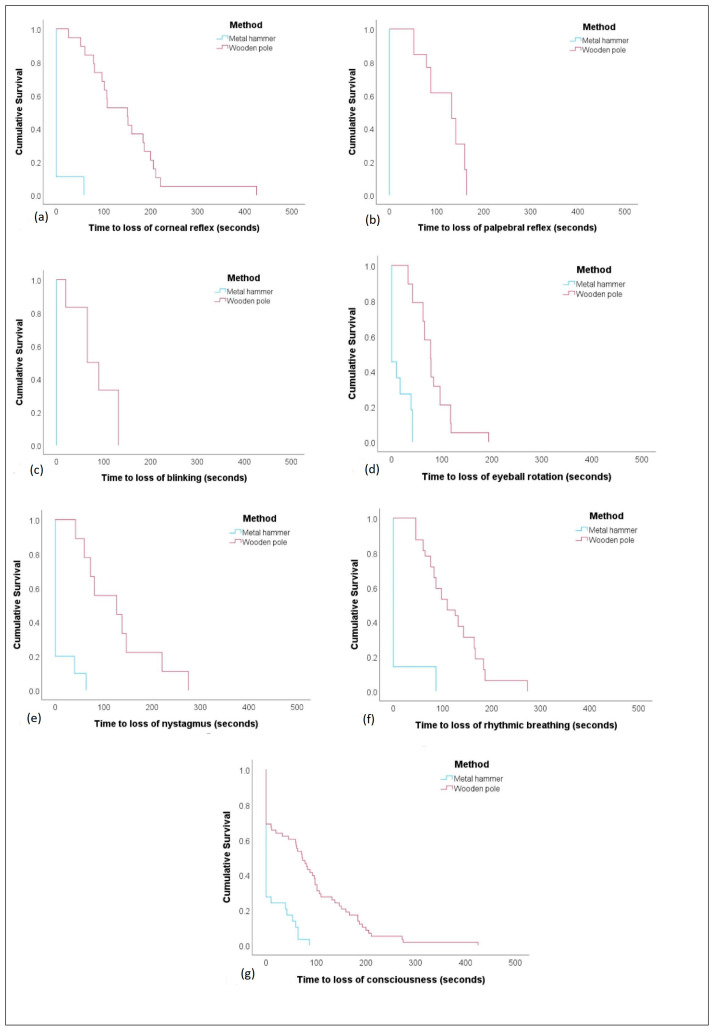
Kaplan–Meier survival curves for time to loss of consciousness signs according to BFT methods (metal hammer and wooden pole); (**a**) corneal reflex, (**b**) palpebral reflex, (**c**) blinking, (**d**) eyeball rotation, (**e**) nystagmus, (**f**) rhythmic breathing, and (**g**) loss of consciousness, i.e., loss of all above signs.

Survival analysis found a significant difference (*p* ≤ 0.001) in time to loss of consciousness between the wooden pole and metal hammer BFT methods, as well as for each individual sign (corneal reflex, palpebral reflex, blinking, eyeball rotation, nystagmus, and rhythmic breathing) (Figure 3).

**Figure 4:**
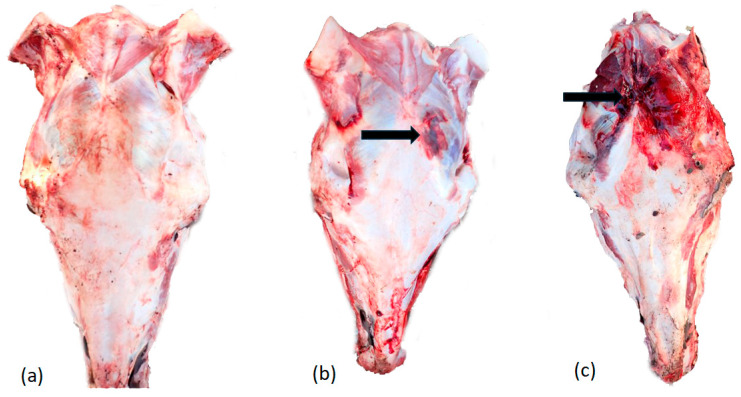
Examples of donkey heads post-mortem (and post-skinning) after BFT: (**a**) head from location C (wooden pole) without any obvious macroscopic haemorrhage or impact site but with radiating fractures; (**b**) head from location D (metal hammer) with impact site/haemorrhage indicated with arrow, (**c**) head from location E (metal hammer) with significant haemorrhage, radiating fractures and impact site indicated with arrow.

For the subset of animals where heads were examined (but could not be matched to animal assessed at slaughter), skull fractures were most prevalent in donkeys subject to BFT by metal claw hammer at either location D or E. Fractures were present in 86% (25/29) of animals hit with a hammer and in 26% (6/23) of animals hit with the wooden pole. Surface bruising/haemorrhage was present in 76% (22/29) of animals hit with the metal hammer and 48% (11/23) of animals hit with the wooden pole (Figure 4).

**Figure 5:**
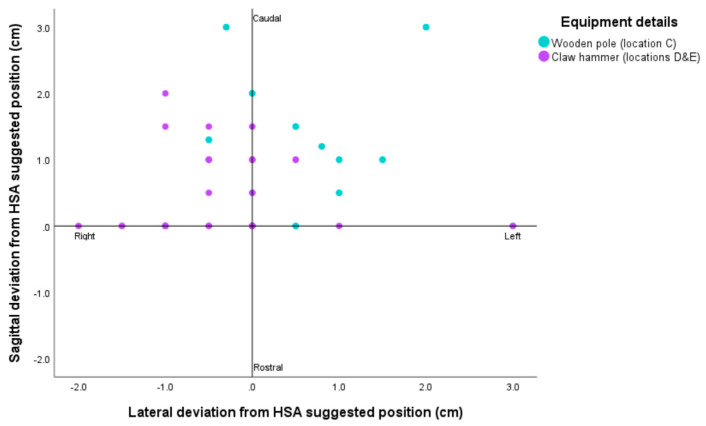
Examination of donkey heads post-mortem after BFT and deviation from suggested position (Humane Slaughter Association: 20mm above where corner of eyes and ears cross). N.B. Heads had been skinned prior to examination.

Where impact site was able to be examined, heads were measured for deviation from the HSA’s suggested position for stunning/slaughter (Figure 5), with all impacts caudal to the suggested position.

Linear regression found a significant association for the time taken from first hit to first cut and animals who showed signs of a return to consciousness (*p* < 0.001). Location C also made more cuts to the neck (7.7 ± (SE) 0.6, range 2–22) in comparison to locations D and E (*p* = 0.001).

For VNI post-BFT at all locations, knives were sharpened on other knives between each animal rather than purpose-built sharpening devices (knife steel or similar), and operators were not seen to clean the knife between animals. Knives at location C were assessed as being in a poor condition, with locations D and E assessed as being moderate. Locations C and E had ≥ 5 nicks and location D had < 5 nicks. Location C had a blade length of 189mm, location D was 187 mm, and location E was 314 mm.

At location C, 71% (41/58) of animals had neck cut placement at position 0, whilst 24% (14/58) were cut at the first trachea ring. At location D/E, 90% were cut at position 0 with 3% cut at position 1. One animal at location C and two animals at location D/E did not have the right carotid artery severed from the cut. None of these showed a return to consciousness. All animals had severed jugular veins (left and right) and severed left carotid arteries. Clotting was absent for all animals.

False aneurysms were observed at both location C (14%; 8/58) and locations D and E (17%; 5/29) (Table 11), with a higher neck cut (at position 0/larynx) significantly more likely to result in false aneurysm (*p* < 0.001, OR 4.5, 95% CI: 2.4–8.4) compared to cuts made at trachea ring 1 or below. The number of neck cuts was not significantly associated with false aneurysms (*p* = 0.13).

### 3.3. Questionnaire

Fifteen abattoir personnel were interviewed, two each from locations A and B, and three each from locations C, D, and E. The mean length of service as slaughter personnel was 14 years (± SD 9.2), with most interviewees having worked in their role since childhood (aged ≥8 years) and learned the trade from their elders or more experienced colleagues.

The majority (87%; 13/15) had experienced injuries through performing the job, ranging from mild knife cuts to kicks from animals and severe cuts or bone chips. Despite this, 87% (13/15) of interviewees deemed the practice either moderately safe or very safe, with only 13% (2/15) deeming it unsafe. They listed safety concerns including the use of blunt knives and the strength of some (particularly male) donkeys and felt that some risks could be mitigated by Personal Protective Equipment and sharper knives or machinery to assist. There was no significant association between accident occurrence and safety classification (*p* = 0.58).

All interviewees felt that the method used (either BFT or non-stun VNI) was efficient, although 93% (14/15) said that they would consider alternative methods if one was available. The criteria for assessing effectiveness by abattoir personnel were listed as checking severance of the spinal cord (27%, 4/15), that the animal has collapsed and fails to attempt to right itself (27%, 4/15) and that the donkey has stopped kicking (47%; 7/15).

All interviewees were open to suggestions for improvements, with one quoted as saying “we need and want better” and another saying “if it could be improved that would be good. I don’t know how but happy to use improvements if they were found”. One said, “this is all we know, but our knowledge is evolving, and we would be willing to use other methods if a better one was found”. They mentioned that before they used BFT or before they restrained by holding a hind leg up and flipping the donkey over, they would tie the donkeys’ mouths shut and cast them onto their side, so consider that BFT is already an improvement to previous method.

## 4. Discussion

This study aimed to identify factors affecting the welfare of donkeys being slaughtered in Ghana, both ante-mortem and during different methods of slaughter. Both non-stun ventral neck incision (VNI) and blunt force trauma (BFT) using either a wooden pole or metal hammer were evaluated at five different locations in the Upper East region of Ghana. Across locations, there was a high proportion of negative human–animal interactions and animals showing stress-related behaviours. Multiple attempts were required for BFT, particularly for animals hit with a wooden pole, which appeared particularly ineffective in comparison to the metal hammer. Furthermore, delayed BFT to VNI times resulted in animals returning to consciousness, potentially prolonging suffering for the animals throughout the slaughter process.

In the donkeys assessed in the ante-mortem phase, more than a third were underweight (38%), and 66% presented with lesions, scars, or swellings (66%), corresponding with previous assessment of donkeys in Africa [36,37]. Hobbling to restrict movement and isolation from conspecifics was commonplace at some locations but not at others, preventing analysis of this as any associations with behaviours observed could be location-specific rather than due to these specific factors.

Many donkeys showed little or no reaction to handling methods. However, harsh treatment and frequent beating of donkeys has been reported as commonplace in Africa, where donkeys are used for work [38,39]. This can then affect their behaviour prior to slaughter, where they are already habituated to poor handling and other stressors. In addition, donkeys are notoriously much more complex and subtle in their expression of pain or distress behaviour than horses and commonly display a freeze response, which is more difficult to interpret than more overt signs of flight or fight [40]. The behaviours commonly seen in this study are indicative of previous findings regarding donkeys’ expression of pain, discomfort, or distress [22]. A review into the slaughter of other species suggested that ante-mortem pain and distress can be further exacerbated through inhumane slaughter [41].

BFT has been found to be an ineffective and inconsistent method of slaughter [11,12]. The present study supports this, finding that 43% of animals tried to right themselves post-BFT (with the wooden pole), and it often took multiple attempts at BFT (five attempts for one animal) to achieve immobilisation, with 45% showing signs of being ineffectively concussed. This happened more often for animals receiving BFT by a wooden pole, in comparison to those receiving BFT by metal hammer, with the metal hammer also achieving a quicker time to loss of consciousness.

There were occasions where unconsciousness was transient after BFT, especially for those hit by the wooden pole, with 28% showing return of corneal reflex and 34% showing nystagmus returning after initial loss of consciousness. In one donkey, behavioural and brainstem indices of consciousness/sensibility returned after a period of 254 s after the initial hit and another showed corneal reflex up to 425 seconds’ post-hit. This demonstrates the significant potential of welfare compromise through BFT, due to both the potential of prolonged consciousness following it BFT and the potential for a return to consciousness if exsanguination if not conducted promptly.

Neither of the BFT methods assessed were purpose-built BFT devices, with the wooden pole being lightweight untreated soft wood (it was not possible to weigh the poles used) and makeshift in terms of its use for BFT. These findings suggest that a metal hammer is more capable of dispensing the required concussive force, through transfer of kinetic energy to the skull and brain [42], to successfully stun the animal. However, it requires appropriate mass and velocity, and more strength and skill than purpose-built stunning equipment such as a captive bolt gun (CBG). Time to loss of consciousness after CBG stunning compared to BFT has not been directly assessed in equids. However, studies on CBG stunning suggests that it produces near immediate unconsciousness, that signs of consciousness/recovery are observed far less frequently, and with higher reported rates of successful stunning [20,43,44,45]. The use of penetrating or non-penetrating CBG stunning, as an alternative to manual BFT, is unlikely to be a realistic or feasible option for donkey slaughter operatives in Ghana due to economic restrictions and difficulties obtaining cartridges compared to the relative ease and lack of operational costs for BFT. It would also require training in correct use and maintenance to ensure optimal performance [46].

Pathology to determine depth of concussion or level of brain damage was not conducted in the present study. However, BFT is likely to cause less damage than penetrating or non-penetrating CBG. Concussive BFT is thought to occur through the kinetic energy involved in the rapid acceleration of the device to the skull [47,48]. Therefore, even in cases where BFT did appear to be initially successful in immobilising animals, irrecoverable unconsciousness is not guaranteed, reinforcing the need for a secondary killing method such VNI [42]. A delayed time between BFT and exsanguination was indeed associated with a return of signs of consciousness. In particular, at site C, which utilised the wooden pole, 7% of donkeys had not been bled by 120 s, with an overall mean time of 56.4 s between BFT and exsanguination. Whilst researcher examination of each animal post-BFT could have increased the time it took to then bleed the animals, this seemed to rarely be the case as delays appeared to be caused by multiple factors, including a change in operators for each stage and animals being hobbled to other animals, which began showing signs of ineffective stunning and/or recovery. It is challenging to determine optimal stun-to-stick times based on when signs returned as these times were variable, from 5 s to 254 s. However, for animals slaughtered without stunning, loss of jaw tone, which suggests a loss of muscle control and the onset of unconsciousness [49], was seen in 70% of animals within 50 s. Loss of rhythmic breathing was also seen in 100% of animals within 50 s. Eye reflexes continued for longer, indicating that brainstem function continued through intact cranial nerves post-VNI. For animals slaughtered through BFT, signs of consciousness returned up to 254 s post-BFT (with wooden pole) but a mean time of 65.1 s (31.3 s for those hit with the metal hammer). Therefore, conducting VNI within 50 s of effective stunning with BFT is optimal to minimise recovery risk. 

If the animal is not stunned or ineffectively stunned and is still conscious prior to VNI then, even when it is conducted quickly and cleanly, they can experience stress and pain prior to loss of consciousness, through the severance of sensitive tissues in the neck stimulating nociceptors in these tissues [15,16,17,49,50,51,52]. VNI aims to cause a loss of consciousness through decreasing cerebral blood flow through severance of the blood vessels that supply the brain, depriving it of oxygen [52]. However, for those animals slaughtered by VNI without BFT, it took up to 104 and 164 s to lose muscle tone and corneal reflex, respectively. This delay in losing consciousness following VNI corresponds with Terlouw et al. [53] who found that eye movements were seen to continue in cattle for up to three minutes after bleeding.

Suffering after VNI can be further prolonged when there is impeded blood flow as a result of arterial occlusion or false aneurysms, leading to maintenance of brain function [16,17,54,55]. In this study, false aneurysms were seen in just one non-stunned animal and 13 BFT animals, where 82% animals had VNI made above the trachea rings (position 0). Cuts made at the first or second trachea ring were less likely to be associated with false aneurysm than cuts at the larynx but there were no cases of cuts made below the second trachea ring. Previous studies have found that cuts performed at the first cervical vertebrae reduced false aneurysm prevalence in cattle compared to cuts made lower down the cervical vertebrae [54,56,57]. However, drawing conclusions across species should be avoided as it is possible that donkeys have different neck anatomy and physiology. This could warrant further investigation in future studies.

Efficient bleeding can be increased through sharper knives [49,58], with blunt knives requiring multiple neck cuts to severe the tissues and vessels of the neck and an average of 6.5 cuts made in the present study. This increases pain by affecting more nociceptors [49,51,58,59] and increases constriction, clotting or carotid occlusion through ineffective bleeding [49,58,59]. Whilst knife sharpness was not objectively measured in the present study, all knives were subjectively assessed as being in a poor condition and/or nicked. An affordable way of addressing this issue could be the provision of manual knife sharpeners and replacing knives when they become over-sharpened, especially considering that poor knife conditions can also increase injury risk to personnel [58]. A high proportion of personnel reported injuries in this study, particularly from knives, but there was a distinct lack of personal protective equipment (PPE). This corresponds with cattle slaughterhouses in Nigeria [8] and is likely due to a lack of available funds, resources or awareness of the importance of PPE.

None of the operators surveyed in this study had received any formal training on animal welfare at slaughter, corresponding with other studies of slaughter personnel in Ghana and adjoining West African countries [7,8,36]. Many operators surveyed said that they had been involved in the profession since as young as eight years old, raising further concerns regarding operator fatigue, particularly where child operators seemed to lack the strength required to appropriately ‘tip’ or restrain the donkey prior to non-stun slaughter. Such child labour in Ghanaian agriculture is common, with high numbers of children working in the agricultural industry [60] raising the question of how attitudes and behaviour can be improved when aversive behaviour towards animals is normalised from a young age, at a time when attitude formation is crucial [61].

Slaughter operatives are already under pressure to maximise throughput of donkeys through the slaughter process [6], and a lack of animal welfare knowledge or training, alongside such a high workload, can lead to increased injury risk, compassion fatigue, and decreasing quality of work [59]. Even where knowledge exists in some African countries, welfare improvements are restricted by constrained resources [9] and economic situations prohibiting ethical choices over food, often making animal welfare seen as a ‘luxury’ [62]. However, ante-mortem stress to animals can negatively affect meat quality and food safety by risking pathogens in the meat that could lead to zoonotic infections in humans when consumed [8]. Raising awareness of the economic and public health impacts of mitigating this risk could engage slaughter operatives and policymakers with animal welfare. This would require buy-in from industry stakeholders and targeted interventions to address barriers to change, such as restricted resources, lack of support, and education to improve operator attitudes and behaviour towards animals [63,64].

This study was limited by the sample size from each location, but this was constrained by logistics in the field and limited resources, and a larger sample size is unlikely to have altered the trends seen. Data collected through structured questionnaires were exploratory and did not attempt to generate a deeper understanding of personnel attitudes, but these findings could inform questions for more in-depth interviews in the future. Frequency and duration of ante-mortem animal behaviour was not assessed, and the presence of behaviours were recorded in different time windows, with ante-mortem indicators not linked to the outcome for animal, due to logistical/resource restrictions. Data collection was also impeded for a small proportion of animals sampled by visibility, resulting in some missing data, with slaughter being conducted during hours of darkness at some locations, without sufficient artificial lighting to assess individual animals at a distance, and subsequently restricting observations of animal health and behavioural parameters. Signs of consciousness/unconsciousness were more readily observable, as this was performed at close range rather than at a distance, with a torch when required. However, the environment of location B was significantly darker complicating assessment of rhythmic breathing through ribcage movement. The complications with lighting conditions meant that some data were missing, but the research team were generally able to examine animals separately with the assistance of torchlight and any missing data are not considerable as to affect any commonalities seen amongst animals. However, as this was also usual practice for these locations, it is an important consideration when evaluating the effectiveness of slaughter in such situations, with this hindering the slaughter operator’s ability to see positioning of either knife or blunt force trauma instrument to slaughter most proficiently. To our knowledge, this is the first study of its kind in Ghana, providing an initial assessment of donkey welfare during slaughter using a range of methods, and being representative of a standard slaughter environment in West Africa, these limitations are to be expected from a field-based, exploratory study, and do not detract from the central findings.

## 5. Conclusions

In conclusion, the welfare of donkeys at slaughter in Ghana is compromised both ante-mortem, through negative human–animal interactions, involving forceful handling and impeded movement, and at slaughter. BFT using a wooden pole has variable and limited success with achieving irrecoverable concussion. For non-stun VNI, eye reflexes and muscle tone continued for up to 164 s and 104 s, respectively. Such signs of consciousness suggest animals still have functioning brain activity and the ability to suffer could be prolonged by experiencing distress and pain. The lack of welfare of donkeys at slaughter in Ghana is, therefore, multifactorial and requires a holistic approach to facilitate simple and realistic improvements. The findings of this study can be used as evidence for policymakers and to support slaughter personnel in Ghana and other countries to make incremental improvements in their treatment of donkeys at slaughter.

## Figures and Tables

**Table 1:** Behavioural ethogram used to assess donkeys during movement from holding pen to slaughter area and restraint prior to slaughter.

Behaviour	Description	Reference
Standing on 4 feet	Weight-bearing on all four limbs with no preferred loading	Regan et al., 2014 [22]
Bite/bite threat	Grasps object, self, or another donkey/horse with open mouth and bites or chews with single or repeated jaw closures	Regan et al., 2014 [22]
Tail tuck	Tail held tightly against rump in fixed position, with tip of tail tucked between hind legs.	Regan et al., 2014 [22]
Lying sternally	Lying down on sternum, legs folded underneath body frame	Regan et al., 2014 [22]
Lying laterally	Lying on side with legs outstretched; head and neck may be in contact with ground	Regan et al., 2014 [22]
Vocalisation	Braying (series of short duration, loud inhalations, followed by a prolonged noisy exhalation)	Regan et al., 2014 [22]
Snort	Prolonged noisy exhalation	Regan et al., 2014 [22]
Refuse to walk	The donkey stops moving, digging in its heels, refusing to move	Dai et al., 2020 [23]
Rear	The donkey rears with front legs	Dai et al., 2020 [23]
Kick	The donkey kicks, one or two legs are lifted and moved rapidly and forcefully	Dai et al., 2020 [23]
Defecate	The donkey drops manure	Dai et al., 2020 [23]
Urinate	The donkey drops urine	Dai et al., 2020 [23]
Paw	The donkey raises a foreleg and scrapes the floor	Dai et al., 2020 [23]
Sniffing	The donkey sniffs the ground	Dai et al., 2020 [23]
Increased respiration	The donkey breathes rapidly, greater than is characteristic (>12 to 28 breaths/minute)	Ayala et al., 2021 [24]
Yawning	Frequent bouts of yawning, often with a greater number of yawns per bout than is characteristic (greater than three to five)	Torcivia & McDonnell, 2021 [25]
Tail swishing	Moving tail suddenly from side to side, similar to that seen in response to cutaneous irritation, e.g., insects.	Torcivia & McDonnell 2021 [25]
Dull/depressed demeanour	Less responsive to the environment, often with a “zoned out”, worried, or glassy-eyed staring facial expression.	Torcivia & McDonnell 2021 [25]
Ears back	Rotating ears to focus caudally or laying ears back against neck	Torcivia & McDonnell 2021 [25]
Head shaking	Rotational shaking of the head	Torcivia & McDonnell 2021 [25]
Weight shifting	Frequent shifting of the primary weight-bearing limb(s)	Torcivia & McDonnell 2021 [25]
Licking/chewing	Moving jaw or moving tongue around their mouth and lips	Jaeger, 2017 [26]
Trembling	Trembling, shivering, or shaking	Pearson et al., 2021 [27]
High blink rate	Closing movement of the eyes, greater than is characteristic (greater than once per 5 s)	Fenner et al., 2016 [28]
Slip	A loss of balance, without any part of the body (other than hooves) touching the ground	Felici et al., 2022 [29]
Fall	A loss of balance, causing any part of the body (other than hooves) to touch the ground	Felici et al., 2022 [29]

**Table 2:** Human–animal interactions during movement from holding pen to slaughter area and restraint prior to slaughter.

Indicator	Category	Description	Reference
Reaction to operator	Calm/alert Avoidant/nervous Aggressive/agonistic Apathetic/depressed	Turns head towards/ears forward Moves or attempts to move away/turns head away Attempts to bite, rear, kick, strike; ears are back or flattened Passive response to surroundings, e.g., head lowered	Burn et al. (2010) [30]
Personnel vocalisations	Speaking Shouting Rattling/slamming Nothing of note	Speaks or whistles softly/quietly Speaks or shouts harshly/loudly Makes noise by clapping hands, slamming wall, etc.	Hultgren et al. (2014) [31]
Personnel attitude	Positive Negative Neutral	Talking quietly, petting, touching Talking/shouting impatiently, forceful use of stick/hand Dominant talking, gentle touch with stick or hand	Waiblinger et al. (2002) [32]
Equipment used	Flat metal object Weighted/pointed metal object Wooden object Kick with foot Hit with hand None	E.g. knife, machete E.g. claw hammer E.g. stick, wooden pole	N/A
Manner in which equipment used	Gentle Intense Rough	Soft and/or <5x Stronger than before without damaging and/or >5–<10x Excessive force, damaging and/or >10x	Huertas et al. (2018) [33]

**Table 3:** Brainstem and behavioural signs of insensibility/unconsciousness assessed immediately post-stun or post-cut and repeatedly approximately every five seconds until all signs were absent.

Behaviour/Signs	Description
Lack of immediate collapse	Animal fails to collapse immediately after the hit
Righting reflex	Makes co-ordinated effort to stand or lift head
Palpebral reflex	Involuntary blink reflex when the medial canthus is stimulated
Corneal reflex	Involuntary blink reflex when cornea is stimulated
Nystagmus	Rapid involuntary movements of the eye
Eyeball rotation	Eyes rotated, not central, sclera visible
Spontaneous blinking	Opens/closes eyelid without stimulation
Gasping *	Spasmodic sharp intake of breath with the mouth open
Nostrils flaring *	Movements/trembling of the nostrils
Rhythmic breathing	Ribcage continuously moves in and out rhythmically
Absence of tonic convulsions Absence of clonic convulsions	Stiffening/rigidity of muscles Uncontrolled involuntary kicking movements
Muscle spasms	Absence of tonus in body/excessive muscle activity
Vocalisation *	Vocalises independently from exhalation
Jaw tone	Absence of a relaxed jaw/protruding tongue

* Reported when observed. Other behaviours listed in order of observation.

**Table 4:** Frequency of human–animal interactions observed at each slaughter point.

Human–Animal Interactions	Location A	Location B	Location C	Location D	**Location E**	**TOTAL**
Personnel vocalisations -Speaking -Shouting -Rattling/slamming -Nothing of note	24% (10/41) 76% (31/41) 0% (0/41) (0/41)	0% (0/12) 100% (12/12) 0% (0/12) 0% (0/12)	0% (0/54) 96% (52/54) 0% (0/54) 4% (2/54)	67% (6/9) 11% (1/9) 11% (1/9) 11% (1/9)	30% (6/20) 10% (2/20) 0% (0/20) 60% (12/20)	16% (21/135) 73% (98/135) 1% (1/135) 11% (15/135)
Personnel attitude -Positive -Neutral -Negative	0% (0/41) 0% (0/41) 100% (41/41)	0% (0/12) 0% (0/12) 100% (12/12)	0% (0/54) 4% (2/54) 96% (52/54)	0% (0/9) 11% (1/9) 89% (8/9)	0% (0/20) 0% (0/20) 100% (20/20)	0% (0/135) 2% (3/135) 98% (132/135)
Separated from conspecifics for slaughter? -Yes -No	0% (0/41) 100% (41/41)	100% (12/12) 0% (0/12)	7% (4/54) 93% (50/54)	100% (9/9) 0% (0/9)	90% (18/20) 10% (2/20)	32% (43/135) 68% (92/135)

**Table 5:** Equipment used at each slaughter point including maximum number of times it was used on an animal and/or at a location in total (Note some animals scored multiple types of equipment and/or hit).

Equipment Used	Location A (*n* = 41)	Location B (*n* = 12)	Location C (*n* = 54)	**Location D** **(*n* = 9)**	**Location E** **(*n* = 20)**	**Total (Per Equipment)**
Flat metal object (knife/machete) -Gentle -Intense -Rough	12% 0% 4% 7%	100% 58% 42% 58%	56% 0% 4% 5%	22% 0% 11% 22%	15% 5% 0% 10%	Total: 27 animals, Median: 3.5 (IQR: 3.75; 1–30) 10 animals; Median: 3 (IQR: 3.25; 1–12) 10 animals; Median: 2.5 (IQR: 1.0; 2–4) 18 animals;Median: 3 (IQR: 3.5; 1–30)
Wooden object (stick/pole) -Gentle -Intense -Rough	41% 7% 15% 39%	0% 0% 0% 0%	85% 19% 37% 83%	44% 0% 33% 34%	70% 15% 35% 80%	Total: 86 animals, Median: 7 (IQR: 6; 1–23) 16 animals; Median: 2 (IQR: 1.5; 1–12) 38 animals; Median: 3 (IQR: 2; 1–13) 75 animals; Median: 6.5 (IQR: 5.75; 1–23)
Hit with hand -Gentle -Intense -Rough	22% 0% 10% 7%	0% 0% 0% 0%	4% 2% 2% 2%	22% 11% 0% 22%	10% 0% 0% 10%	Total: 13 animals, Median: 3 (IQR: 2; 1–11) 1 animal < 1× 3 animals; Median: 4 (IQR: 4.5; 2–11) 5 animals; Median: 3 (IQR: 2; 1–5)
Other -Leg of other (dead) donkey -Rope (whipped)	2% 0%	0% 0%	0% 0%	0% 33%	0% 10%	1 animal < 7× 5 animals < 12×

**Table 6:** Correlations between behaviours determined through Kendall’s tau-b correlation (positive correlations in bold).

Behaviour	Behaviour								
	Tail Tuck	Rapid Breathing	Trembling	Blinking *	Refuse to Walk	Sniffing Ground	**Tail Swishing**	**Chew/Jaw Movement**	**Ears Flat Back**
Tail tuck	-	**τ_b_ = 0.300** ***p* < 0.001**	τ_b_ = −0.018 *p* = 0.84	**τ_b_ = 0.283** ***p* = 0.001**	**τ_b_ = 0.233** ***p* = 0.007**	τ_b_ = −0.065 *p* = 0.45	τ_b_ = −0.165 *p* = 0.08	τ_b_ = 0.042 *p* = 0.65	**τ_b_ = 0.212** ***p* = 0.02**
Rapid breathing	**τ_b_ = 0.300** ***p* < 0.001**	-	τ_b_ = 0.157 *p* = 0.07	τ_b_ = 0.545 ***p* < 0.001**	τ_b_ = 0.086 *p* = 0.32	τ_b_ = 0.049 *p* = 0.57	τ_b_ = 0.073 *p* = 0.43	τ_b_ = 0.173 *p* = 0.06	**τ_b_ = 0.339** ***p* = 0.001**
Trembling	τ_b_ = −0.018 *p* = 0.84	τ_b_ = 0.157 *p* = 0.07	-	**τ_b_ = 0.244** ***p* = 0.005**	τ_b_ = −0.029 *p* = 0.74	τ_b_ = 0.002 *p* = 0.99	**τ_b_ = 0.258** ***p* = 0.005**	τ_b_ = 0.131 *p* = 0.16	τ_b_ = −0.008 *p* = 0.93
Blinking *	**τ_b_ = 0.283** ***p* = 0.001**	**τ_b_ = 0.545** ***p* < 0.001**	**τ_b_ = 0.244** ***p* = 0.005**	-	τ_b_ = 0.122 *p* = 0.16	τ_b_ = 0.130 *p* = 0.13	τ_b_ = 0.165 *p* = 0.08	**τ_b_ = 0.262** ***p* = 0.005**	**τ_b_ = 0.442** ***p* < 0.001**
Refuse to walk	**τ_b_ = 0.233** ***p* = 0.007**	τ_b_ = 0.086 *p* = 0.32	τ_b_ = −0.029 *p* = 0.74	τ_b_ = 0.122 *p* = 0.16	-	τ_b_ = −0.075 *p* = 0.39	τ_b_ = 0.008 *p* = 0.93	τ_b_ = 0.057 *p* = 0.54	**τ_b_ = 0.215** ***p* = 0.02**
Sniffing ground	τ_b_ = −0.065 *p* = 0.45	τ_b_ = 0.049 *p* = 0.57	τ_b_ = 0.002 *p* = 0.99	τ_b_ = 0.130 *p* = 0.13	τ_b_ = −0.075 *p* = 0.39	-	τ_b_ = 0.135 *p* = 0.15	τ_b_ = 0.138 *p* = 0.14	τ_b_ = 0.046 *p* = 0.61
Tail swishing	τ_b_ = −0.165 *p* = 0.08	τ_b_ = 0.073 *p* = 0.43	**τ_b_ = 0.258** ***p* = 0.005**	τ_b_ = 0.165 *p* = 0.08	τ_b_ = 0.008 *p* = 0.93	τ_b_ = 0.135 *p* = 0.15	-	**τ_b_ = 0.196** ***p* = 0.04**	τ_b_ = −0.133 *p* = 0.16
Chew/jaw movement	τ_b_ = 0.042 *p* = 0.65	τ_b_ = 0.173 *p* = 0.06	τ_b_ = 0.131 *p* = 0.16	**τ_b_ = 0.262** ***p* = 0.005**	τ_b_ = 0.057 *p* = 0.54	τ_b_ = 0.138 *p* = 0.12	**τ_b_ = 0.196** ***p* = 0.04**	-	τ_b_ = 0.398 ***p* = 0.001**
Ears flat back	**τ_b_ = 0.212** ***p* = 0.02**	τ_b_ = 0.339 ***p* = 0.001**	τ_b_ = −0.008 *p* = 0.93	τ_b_ = 0.442 ***p* = 0.001**	**τ_b_ = 0.215** ***p* = 0.02**	τ_b_ = 0.046 *p* = 0.61	τ_b_ = −0.133 *p* = 0.16	τ_b_ = 0.398 ***p* = 0.001**	-

* Not assessed in some animals due to poor lighting conditions.

**Table 7:** Behavioural indicators logged for donkeys at each slaughter point location, and one-way Anova test with Tukey–Kramer post hoc test for differences between locations for each behaviour.

	Location					*p*-Value
	A	B	C	D	E	
Rapid breathing	86% (30/35) ^B^	75% (9/12) ^ACDE^	100% (54/54) ^B^	100% (9/9) ^B^	100% (20/20) ^B^	<0.001
Spontaneous blinking *	81% (26/32) ^B^	33% (4/12) ^ACDE^	98% (47/48) ^B^	89% (8/9) ^B^	95% (19/20) ^B^	<0.001
Tail tuck	54% (19/35)	75% (9/12)	67% (36/54)	78% (7/9)	100% (20/20)	0.06
Ears flat back	49% (17/35) ^BCDE^	0% (0/12) ^ACDE^	83% (45/54) ^AB^	100% (9/9) ^AB^	65% (13/20) ^AB^	<0.001
Refuse to walk	9% (3/35) ^CDE^	50% (6/12) ^CDE^	35% (19/54) ^AB^	100% (9/9) ^AB^	70% (14/20) ^AB^	<0.001
Trembling	31% (11/35)	58% (7/12) ^E^	35% (19/54)	44% (4/9)	5% (1/20) ^B^	0.03
Sniffing ground	66% (23/35) ^BC^	0% (0/12) ^AE^	70% (7/54) ^AE^	11% (1/9)	35% (7/20) ^BC^	<0.001
Chew/jaw movement	29% (10/35)	0% (0/12)	30% (16/54)	44% (4/9)	10% (2/20)	0.06
Tail swishing	43% (15/35) ^DE^	50% (6/12) ^DE^	20% (11/54)	0% (0/9) ^A^	0% (0/20) ^AB^	<0.001

* Not assessed in some animals due to poor lighting conditions. Columns with different superscripts (A–E) mark which locations differ significantly following the Tukey–Kramer post hoc test, where *p* < 0.05, e.g., rapid breathing at location A was different to location B, whilst rapid breathing at location B was different to locations A, C, D, and E.

**Table 8:** Mean (±SE) time to loss of behavioural and brainstem indices for animals slaughtered by VNI. Differences between sites were tested with a Mann–Whitney independent sample. NB. Time to loss of taken from first cut. Eye movements were assessed from right-hand side.

Brainstem Sign (Time to Loss)	Location A (*n* = 36)	Location B (*n* = 14)	*p*-Value
Attempted rhythmic breathing	16.1 ± 4.8 (*n* = 8) Range 5–46	Absent in all	N/A
Spontaneous blinking	20.7 ± 4.5 (*n* = 7) Range 9–43	Absent in all	N/A
Eyeball rotation	42.7 ± 12.4 (*n* = 13) Range 9–162	29.3 ± 8.1 (*n* = 3) Range 15–43	0.89
Palpebral reflex	47.3 ± 3.1 (*n* = 34) Range 19–96	36.2 ± 3.8 (*n* = 10) Range 18–58	0.09
Jaw tone	44.9 ± 2.9 (*n* = 28) Range 24–104	41.6 ± 4.4 (*n* = 10) Range 23–64	0.51
Corneal reflex	102.3 ± 3.8 (*n* = 36) Range 26–164	109.4 ± 3.3 (*n* = 10) Range 94–124	0.17
Nystagmus	Absent in all	Absent in all	N/A

**Table 9:** Assessment of severance of carotid arteries, jugular veins, and trachea during VNI slaughter, including presence of clotting and/or false aneurysms and location of neck cut (total *n* = 46).

	Location A (*n* = 36)	Location B (*n* = 10)
Right Carotid artery	Severed: 36 Intact: 0	Severed: 10 Intact: 0
Left Carotid artery	Severed: 34 Intact: 2	Severed: 10 Intact: 0
Right jugular vein	Severed: 36 Intact: 0	Severed: 10 Intact: 0
Left jugular vein	Severed: 36 Intact: 0	Severed: 10 Intact: 0
Trachea	Severed: 36 Intact: 0	Severed: 10 Intact: 0
Location of neck cut (trachea rings)	0: 14 1: 15 2: 1 NA: 6	0: 0 1: 8 2: 1 NA: 0
Clotting	Present: 0 Absent: 36	Present: 0 Absent: 10
False aneurysms	Present: 1 Absent: 35	Present: 0 Absent: 10

**Table 10:** Mean (± SE) number of hits and cuts at each BFT location, alongside time from first hit to last hit and last cut and with *p*-value determined through the Mann–Whitney (independent sample) test to measure differences between sites for number of BFT attempts and time from hit to cut.

	Wooden Pole (Location C) (Total n = 58)	Metal Hammer (Locations D&E) (Total n = 29)	*p*-Value
Number of hits per animal ± SE (range)	1.7 ± 0.1 (1–5)	1.3 ± 0.1	0.003
Number of animals hit > 1×	25	5	
Time from first hit to last hit ± SE	24.1 ± 5.6	19.8 ± 10.8	0.27
Time from first hit to first cut ± SE	56.4 ± 6.6	39.9 ± 4.6	0.23
Number of cuts to neck per animal ± SE (range)	7.7 ± 0.6 (2–22)	4.7 ± 0.4 (2–12)	<0.001
Number of cuts to spinal cord per animal ± SE	1.1 ± 0.2	0.6 ± 0.1	0.17

**Table 11:** Assessment of severance of carotid arteries, jugular veins, and trachea during ventral neck incision after blunt force trauma slaughter, including presence of clotting and/or false aneurysms and location of neck cut (total *n* = 87).

	Location C (*n* = 58)	Location D and E (*n* = 29)
Right Carotid artery	Severed: 56 Intact: 1 * NA: 1	Severed: 26 Intact: 2 NA: 1
Left Carotid artery	Severed: 56 Intact: 0 NA: 2	Severed: 27 Intact: 0 NA: 2
Right jugular vein	Severed: 57 Intact: 0 NA: 1	Severed: 28 Intact: 0 NA: 1
Left jugular vein	Severed: 57 Intact: 0 NA: 1	Severed: 28 Intact: 0 NA: 1
Location of neck cut (trachea rings)	0: 41 1: 14 2: 0 NA: 3	0: 26 1: 1 2: 0 NA: 2
Clotting	Present: 0 Absent: 56 NA: 2	Present: 0 Absent: 27 NA: 2
False aneurysms	Present: 8 Absent: 48 NA: 2	Present: 5 Absent: 21 NA: 3

* Vessels that were partially severed were grouped where impeding blood flow with ‘intact’.

## Data Availability

Restrictions apply to the availability of these data. Data were obtained from the participating slaughter points, with appropriate informed consent, and are available from the authors with the permission of the slaughter points.

## Supplementary Materials

The following supporting information can be downloaded at: https://www.mdpi.com/article/10.3390/ani14243673/s1, Questionnaire S1, and Supplementary Table S1 time to loss of consciousness.

## References

[1] (2024). Food and Agricultural Organization Statistical Database: Live Animals. https://www.fao.org/faostat/en/#data.

[2] Bennett R., Pfuderer S. Demand for donkey hides and implications for global donkey populations. Proceedings of the 93rd Annual Conference.

[3] Johnston L.A. (2024). China, Africa, and the Market for Donkeys: Sample of Ejiao’s Bitter Aftertaste in Africa. https://ssrn.com/abstract=4734449.

[4] Norris S.L., Little H.A., Ryding J., Raw Z. (2021). Global donkey and mule populations: Figures and trends. PLoS ONE.

[5] (2024). Donkey Sanctuary. Donkeys in Global Trade. https://www.thedonkeysanctuary.org.uk/sites/default/files/2024-06/donkeys-in-global-trade-wildlife-crime-welfare-biosecurity-and-the-impact-on-women-briefing-2024.pdf.

[6] Maggs H.C., Ainslie A., Bennett R.M. (2023). The value of donkeys to livelihood provision in northern Ghana. PLoS ONE.

[7] Mogre J.W.S., Adzitey F., Teye G.A., Birteeb P.T. (2024). Cattle transporters’ attitudes, indigenous knowledge, and current practices towards animal welfare, occupational well-being, and operational challenges: A survey of five regions in Ghana. Heliyon.

[8] Njoga E.O., Ilo S.U., Nwobi O.C., Onwumere-Idolor O.S., Ajibo F.E., Okoli C.E., Jaja I.F., Oguttu J.W. (2023). Pre-slaughter, slaughter and post-slaughter practices of slaughterhouse workers in Southeast, Nigeria: Animal welfare, meat quality, food safety and public health implications. PLoS ONE.

[9] Lemma M., Mulema A., Kinati W., Wieland B. (2018). Transforming Gender Relations and Reducing Risk of Zoonotic Diseases Among Small Ruminant Farmers in the Highlands of Ethiopia: A Guide for Community Conversation Facilitators.

[10] Ressel L., Hetzel U., Ricci E. (2016). Blunt Force Trauma in Veterinary Forensic Pathology. Vet. Pathol..

[11] Walsh J.L., Percival A., Turner P.V. (2017). Efficacy of blunt force trauma, a novel mechanical cervical dislocation device, and a non-penetrating captive bolt device for on-farm euthanasia of pre-weaned kits, growers, and adult commercial meat rabbits. Animals.

[12] Li X., Zito S., Sinclair M., Phillips C.J.C. (2018). Perception of animal welfare issues during Chinese transport and slaughter of livestock by a sample of stakeholders in the industry. PLoS ONE.

[13] Sinclair M., Hötzel M.J., Lee N.Y.P., de Luna M.C.T., Sharma A., Idris M., Islam M.A., Iyasere O.S., Navarro G., Ahmed A.A. (2023). Animal welfare at slaughter: Perceptions and knowledge across cultures. Front. Anim. Sci..

[14] Terlouw E.C., Le Neindre P. (2024). Consciousness in farm animals and the ‘how’ and ‘why’ of slaughter techniques. Curr. Opin. Behav. Sci..

[15] Gibson T., Johnson C., Murrell J., Mitchinson S., Stafford K., Mellor D. (2009). Electroencephalographic responses to concussive non-penetrative captive-bolt stunning in halothane-anaesthetised calves. N. Z. Vet. J..

[16] Zulkifli I., Goh Y.M., Norbaiyah B., Sazili A.Q., Lotfi M., Soleimani A.F., Small A.H. (2014). Changes in blood parameters and electroencephalogram of cattle as affected by different stunning and slaughter methods in cattle. Anim. Prod. Sci..

[17] Johnson C.B., Mellor D.J., Hemsworth P.H., Fisher A.D. (2015). A scientific comment on the welfare of domesticated ruminants slaughtered without stunning. N. Z. Vet. J..

[18] Fletcher K.A., Limon G., Whatford L.J., Grist A., Knowles T.G., Gibson T.J. (2022). A systematic review of equid welfare at slaughter. Livest. Sci..

[19] World Organisation for Animal Health (2021). Terrestrial Code Online Access—WOAH—World Organisation for Animal Health. Terrestrial Animal Health Code.

[20] Fletcher K.A., Padalino B., Felici M., Bigi D., Limon G., Grist A., Gibson T.J. (2024). Assessment of ante-mortem welfare indicators and the pathophysiology of captive bolt trauma in equids at slaughter. Anim. Welf..

[21] Carroll C.L., Huntington P.J. (1988). Body condition scoring and weight estimation of horses. Equine Vet. J..

[22] Regan F.H., Hockenhull J., Pritchard J.C., Waterman-Pearson A.E., Whay H.R. (2014). Behavioural repertoire of working donkeys and consistency of behaviour over time, as a preliminary step towards identifying pain-related behaviours. PLoS ONE.

[23] Dai F., Mazzola S., Cannas S., Heinzl E.U.L., Padalino B., Minero M., Dalla Costa E. (2020). Habituation to transport helps reducing stress-related behavior in donkeys during loading. Front. Vet. Sci..

[24] Ayala M.D., Carrillo A., Iniesta P., Ferrer P. (2021). Pilot study of the influence of equine assisted therapy on physiological and behavioral parameters related to welfare of horses and patients. Animals.

[25] Torcivia C., McDonnell S. (2021). Equine Discomfort Ethogram. Animals.

[26] Jaeger E.E. (2017). Reward Preferences in Domestic Horses (*Equus caballus*). Master’s Thesis.

[27] Pearson G., Waran N., Reardon R.J.M., Keen J., Dwyer C. (2021). A Delphi study to determine expert consensus on the behavioural indicators of stress in horses undergoing veterinary care. Appl. Anim. Behav. Sci..

[28] Fenner K., Yoon S., White P., Starling M., McGreevy P. (2016). The Effect of Noseband Tightening on Horses’ Behavior, Eye Temperature, and Cardiac Responses. PLoS ONE.

[29] Felici M., Nanni Costa L., Zappaterra M., Bozzo G., Di Pinto P., Minero M., Padalino B. (2022). Journeys, Journey Conditions, and Welfare Assessment of Broken (Handled) Horses on Arrival at Italian Slaughterhouses. Animals.

[30] Burn C.C., Dennison T.L., Whay H.R. (2010). Relationships between behaviour and health in working horses, donkeys, and mules in developing countries. Appl. Anim. Behav. Sci..

[31] Hultgren J., Wiberg S., Berg C., Cvek K., Lunner Kolstrup C. (2014). Cattle behaviours and stockperson actions related to impaired animal welfare at Swedish slaughter plants. Appl. Anim. Behav. Sci..

[32] Waiblinger S., Menke C., Coleman G. (2002). The relationship between attitudes, personal characteristics and behaviour of stockpeople and subsequent behaviour and production of dairy cows. Appl. Anim. Behav. Sci..

[33] Huertas S.M., Kempener R.E.A.M., van Eerdenburg F.J.C.M. (2018). Relationship between Methods of Loading and Unloading, Carcass Bruising, and Animal Welfare in the Transportation of Extensively Reared Beef Cattle. Animals.

[34] HSA. 2013. Captive-Bolt Stunning of Livestock. www.hsa.org.uk.

[35] Gibson T.J., Bedford E.M., Chancellor N.M., Limon G. (2015). Pathophysiology of free-bullet slaughter of horses and ponies. Meat Sci..

[36] Agegnehu A., Abebaw G., Nejash A. (2017). Health and welfare status of donkeys in and around Hawassa Town, Southern Ethiopia. J. Vet. Med. Anim. Health.

[37] Mshelia P.W., Aji H.M., Akinniyi O.O., Edeh R.E. (2023). Welfare Assessment of Pack Donkeys in Amaru, Zaria Ancient City, Kaduna State, Nigeria. Folia Vet..

[38] Chaburte C., Endabu B., Getahun F., Fanta A., Asefa Z., Aragaw K. (2019). Health and welfare problems of pack donkeys and cart horses in and around Holeta town, Walmara district, Central Ethiopia. J. Vet. Med. Anim. Health.

[39] McLean A.K., Heleski C.R., Yokoyama M.T., Wang W., Doumbia A., Dembele B. (2012). Improving working donkey (*Equus asinus*) welfare and management in Mali, West Africa. J. Vet. Behav. Clin. Appl. Res..

[40] Haines A., Goliszek J. (2019). Donkey and mule behaviour for the veterinary team. UK VET Equine.

[41] Dalla Costa F.A., Gibson T.J., Oliveira S.E.O., Gregory N.G., Faucitano L., Dalla Costa O.A. (2021). On-farm culling methods used for pigs. Anim. Welf..

[42] Hing S., Hampton J.O., Gibson T.J. (2019). Animal welfare and the killing of wildlife by captive bolt in Australia. Aust. Zool..

[43] Flint M., Sagrera K., Wainwright K., Flint J.B. (2023). Field Based Assessment of Clinical Signs of Irreversible Loss of Consciousness and Death Confirmed by Brain Destruction in Juvenile American Alligators *(Alligator mississippiensis)* After Penetrating Captive Bolt Stunning or Electrostunning with Probe Pithing. J. Appl. Anim. Welf. Sci..

[44] Caraves M., Gallo C. (2007). Characterization and evaluation of the stunning systems used for horses in Chile. Arch. Med. Vet..

[45] Gibson T.J., Whitehead C., Taylor R., Sykes O., Chancellor N.M., Limon G. (2015). Pathophysiology of penetrating captive bolt stunning in Alpacas (*Vicugna pacos*). Meat Sci..

[46] Gibson T.J., Mason C.W., Spence J.Y., Barker H., Gregory N.G. (2014). Factors Affecting Penetrating Captive Bolt Gun Performance. J. Appl. Anim. Welf. Sci..

[47] Sussman E.S., Pendharkar A.V., Ho A.L., Ghajar J. (2018). Mild traumatic brain injury and concussion: Terminology and classification. Handb. Clin. Neurol..

[48] Oliveira S.E.O., Dalla Costa F.A., Gibson T.J., Costa O.A.D., Coldebella A., Gregory N.G. (2018). Evaluation of brain damage resulting from penetrating and non–penetrating stunning in Nelore Cattle using pneumatically powered captive bolt guns. Meat Sci..

[49] Velarde A., Dalmau A. (2017). Slaughter without stunning. Advances in Agricultural Animal Welfare: Science and Practice.

[50] Gibson T.J., Johnson C.B., Murrell J.C., Hulls C.M., Mitchinson S.L., Stafford K.J., Johnstone A.C., Mellor D.J. (2009). Electroencephalographic responses of halothane-anaesthetised calves to slaughter by ventral-neck incision without prior stunning. N. Z. Vet. J..

[51] Mellor D.J., Gibson T.J., Johnson C.B. (2009). A re-evaluation of the need to stun calves prior to slaughter by ventral-neck incision: An introductory review. New Zealand Vet. J..

[52] Johnson C.B., Gibson T.J., Stafford K.J., Mellor D.J. (2012). Pain perception at slaughter. Anim. Welf..

[53] Terlouw E.M., Bourguet C., Deiss V., Mallet C. (2015). Origins of movements following stunning and during bleeding in cattle. Meat Sci..

[54] Bozzo G., Bonerba E., Barrasso R., Roma R., Luposella F., Zizzo N., Tantillo G. (2020). Evaluation of the occurrence of false aneurysms during halal slaughtering and consequences on the animal’s state of consciousness. Animals.

[55] Von Holleben K., Von Wenzlawowicz M., Gregory N., Anil H., Velarde A., Rodriguez P., Goga B.C., Catanese B., Lambooij B. (2010). Report on Good and Adverse Practices-Animal Welfare Concerns in Relation to Slaughter Practices from the Viewpoint of Veterinary Sciences. Report. www.bsi-schwarzenbek.de.

[56] Gibson T.J., Dadios N., Gregory N.G. (2015). Effect of neck cut position on time to collapse in halal slaughtered cattle without stunning. Meat Sci..

[57] Gregory N.G., Von Wenzlawowicz M., Von Holleben K., Fielding H.R., Gibson T.J., Mirabito L., Kolesar R. (2012). Complications during shechita and halal slaughter without stunning in cattle. Anim. Welf..

[58] Kumar P., Abubakar A.A., Imlan J.C., Ahmed M.A., Goh Y.M., Kaka U., Idrus Z., Sazili A.Q. (2023). Importance of Knife Sharpness during Slaughter: Shariah and Kosher Perspective and Scientific Validation. Animals.

[59] Imlan J.C., Kaka U., Goh Y.M., Idrus Z., Awad E.A., Abubakar A.A., Ahmad T., Quaza Nizamuddin H.N., Sazili A.Q. (2020). Effects of slaughter knife sharpness on blood biochemical and electroencephalogram changes in cattle. Animals.

[60] Zdunnek G. (2009). Food and Agriculture Organization of the United Nations. Child Labour and children’s Economic Activities in Agriculture in Ghana.

[61] Kellert S.R., Fox M.W., Mickley L.D. (1984). Attitudes toward animals: Age-related development among children. Advances in Animal Welfare Science 1984/85.

[62] Sazili A.Q., Kumar P., Hayat M.N. (2023). Stunning Compliance in Halal Slaughter: A Review of Current Scientific Knowledge. Animals.

[63] Noah S.D. (2021). Prevalence of Body Injuries and Handling Practices for Slaughter Pigs and Their Association with Meat Quality in Kiamu County, Kenya. Master’s Thesis.

[64] Wilhelmsson S., Hemsworth P.H., Andersson M., Yngvesson J., Hemsworth L., Hultgren J. (2024). Training of transport drivers improves their handling of pigs during loading for transport to slaughter. Animal.

