# Supplementary material for: Assessment of Donkey (Equus asinus) Welfare at Slaughter in Ghana
Source: Animals (Basel). 2024 Dec 19;14(24):3673. doi: 10.3390/ani14243673 (PMC11672695; doi:10.3390/ani14243673)
Supplement: Supplementary file 1 [file animals-14-03673-s001.zip › animals-3335146-supplementary.pdf]

SURVEY OF EQUID SLAUGHTER

This PhD project aims to investigate the welfare of equids at slaughter across the world. The project will consist of welfare assessment at different stages of the slaughter process. As part of this study, we are seeking information from abattoir personnel and industry stakeholders on current practices and management and about their attitudes and behaviour regarding equid slaughter, including where they feel welfare is promoted or compromised during each stage. This information will be gained through participation in a standardised questionnaire.

We are grateful for as much information as you can provide, but you can decline to answer a specific question, at any time if you wish. Participation in the interview will be taken as consent to use the data provided for the purposes of this research. Any information provided will be kept anonymous and stored securely in accordance with the Data Protection Act 2018 (UK). This also means that the participant's workplace will not be identifiable in any reports. Data will be accessible only by Kate Fletcher (PhD student) and Dr Troy Gibson (Supervisor) at Royal Veterinary College, London UK. The data from this interview will be used ONLY for this specific project and any publications or presentations that it may yield, in which only anonymous summaries of data will be presented. Ethics approval has been given by the Social Science Research Ethical Review Board at the Royal Veterinary College (reference URN: **SR2022-0059**).

We emphasize that the aim of this interview is to obtain reliable and up-to-date data. So where possible please use real data, however if an estimate is provided, please indicate this.

Please note, these questions apply ONLY TO HORSES, DONKEYS AND MULES.

Unless otherwise specified, please try to answer by using the data for last year. If not possible, please specify the year for which you are providing data.

For further information on this project or any other queries please contact:

Kate Fletcher

Royal Veterinary College

Hawkshead Lane, North Mymms

Hatfield, Hertfordshire, UK

Kfletcher20@rvc.ac.uk. Tel: 01707 666333

DRAFT

CONSENT FORM

I, the undersigned, consent to participating in this study. I acknowledge that my answers will be analysed and observed for research purposes. I have understood the description of this study above and I have been given the opportunity to ask questions, which have been answered to my satisfaction. I understand that participation in the study is entirely voluntary, and I have the right to withdraw at any time during the interview.

I understand my rights under data protection laws and that I will not be referred to by name/company or otherwise identified in any report or publication. I agree that data gathered in this study may be used for future research, publications and presentations.

Signed: ____

Company/organization: ____

Length of time at abattoir/in position: ________________________________

Function/Position: ____

☐ Plant/farm manager/owner

☐ Operator/technician (state area responsible, e.g. stunning/lairage/hoisting………………………………………….)

☐ Animal welfare officer

☐ Official Veterinarian

☐ Other (please specify role) ______________________

DRAFT

1. EQUIDS SLAUGHTERED

1.1 What are the total annual numbers of equids slaughtered in this plant?

	HORSES numbers real/estimated	DONKEYS numbers real/estimated	MULES numbers real/estimated
Last year			

1.2 Are other species also slaughtered at this plant?

( ) No, just equids ( ) Cattle ( ) Sheep ( ) Swine ( ) Other: ____________________

1.3 How often do you slaughter equids?

( ) Daily ( ) Weekly ( ) Fortnightly ( ) Monthly

1.4 What method of slaughter do you use (for equids)?

( ) Free bullet rifle: Make/Calibre/Ammunition: ________________________________
( ) Free bullet pistol: Make/Calibre/Ammunition: _____________________________________
( ) Captive bolt gun (penetrative): Make/Calibre/Ammunition: ___________________________
( ) Captive bolt gun (non-penetrative): Make/Calibre/Ammunition: ____________________________
( ) Knife: Type:_________________
( ) Striking the head with a mallet
( ) Other. Description:___________________________

1.5 Do you feel comfortable in perform the slaughter of equids using this methods? ( ) Yes ( ) No
Why?____

DRAFT

____

______

1.6 What is your personal opinion about the efficiency of the slaughter method used??

( ) Inefficient – Animal still presenting response to evoked responses.
( ) Reasonably efficient - In some cases, animals present responses to evoked responses.
( ) Efficient - Animal does not respond to evoked responses.
Comments: ____
____
____

1.7 Have you ever had any accident during the performing of the slaughter/stunning methods used in your routine? ( ) Yes ( ) No

Description:____
____
____

1.8 In your experience, how do you classify the method used for slaughter/stunning for the operator safety?

( ) Low safety ( ) moderately safe ( ) Very safe

1.9 Would you like to use other method for slaughter/stunning? What method would you like? Why?

( ) No ( ) Yes Method:_________________________________

Comments: ____
____
____

DRAFT

1.10 Based on your experience, could you outline some of the issues with stunning/slaughter systems you have used which could be addressed? Please answer in the provided box below.

STUNNING/RESTRAINING SYSTEMS	MAJOR CONCERNS/PROBLEMS

DRAFT

1.1 What criteria are most used at your abattoir for each species to evaluate the effectiveness of stunning or slaughter in equids?

Please indicate by circling the relevant words. When not applicable to your case, please tick the "N/A" box. In such cases where answer is "Not at all", please specify the reason for not using them in the column "Reason". In such cases where answer is unknown, please leave blank. For example:

	Not at all*	Very occasionally	Sometimes	Often	Always	N/A	*Reason
Tonic seizures (constant rapid body tremors)	Horses	Horses	Horses	Horses	Horses		
	Donkeys	Donkeys	Donkeys	Donkeys	Donkeys		
Rigidly extended legs	Horses	Horses	Horses	Horses	Horses		
	Donkeys	Donkeys	Donkeys	Donkeys	Donkeys		
Spontaneous blinking	Horses	Horses	Horses	Horses	Horses		
	Donkeys	Donkeys	Donkeys	Donkeys	Donkeys		
No rhythmic breathing immediately after the stun	Horses	Horses	Horses	Horses	Horses		
	Donkeys	Donkeys	Donkeys	Donkeys	Donkeys		
Corneal reflex	Horses	Horses	Horses	Horses	Horses		
	Donkeys	Donkeys	Donkeys	Donkeys	Donkeys		
Palpebral reflex	Horses	Horses	Horses	Horses	Horses		
	Donkeys	Donkeys	Donkeys	Donkeys	Donkeys		
Righting reflex	Horses	Horses	Horses	Horses	Horses		
	Donkeys	Donkeys	Donkeys	Donkeys	Donkeys		
Eyeball rotation	Horses	Horses	Horses	Horses	Horses		
	Donkeys	Donkeys	Donkeys	Donkeys	Donkeys		
Nystagmus[1]	Horses	Horses	Horses	Horses	Horses		
	Donkeys	Donkeys	Donkeys	Donkeys	Donkeys		
Other, please specify:	Horses	Horses	Horses	Horses	Horses		
	Donkeys	Donkeys	Donkeys	Donkeys	Donkeys		

[1] involuntary movement of the eye

DRAFT

2. ADDITIONAL DETAILS REGARDING TRAINING IN EQUID SLAUGHTER DISPATCH METHODS

1.1 Please indicate what type of training you have received from this plant:

____

____

____

____

If you are interested in obtaining a summary of the final results from this study, please leave your contact details below. Your contact details will not be used for any other purpose than provision of this summary unless permission is granted by ticking the box below.

☐ **I would like to receive a summary of results.**

☐ **I am happy to be contacted on this email / telephone number / address for further research associated with this project**

____

____

____

____

____

End.
Thank you for your participation

DRAFT
