# Supplementary material for: Assessment of Donkey (Equus asinus) Welfare at Slaughter in Ghana
Source: Animals (Basel). 2024 Dec 19;14(24):3673. doi: 10.3390/ani14243673 (PMC11672695; doi:10.3390/ani14243673)
Supplement: Supplementary file 1 [file animals-14-03673-s001.zip › animals-3335146-supplementary-tabs1.pdf]

Table S1: Last reported time to loss of consciousness signs for all animals post blunt force trauma (taken from first hit attempt, eye movements assessed from right-hand side), with mean time in seconds ± SE and range in parentheses

Location		Righting reflex	Palpebral reflex	Corneal reflex	Blinking	Nystagmus	Eyeball rotation	Attempted Rhythmic breathing	Jaw tone
C	**Mean time to return (all)**	28.63 ± 11.09 (5-102)	73.25 ± 18.00 (39-124)	61.33 ± 11.78 (26-209)	64.55 ± 11.71 (20-132)	92.27 ± 21.31 (27-254)	57.80 ± 8.38 (10-157)	62.92 ± 10.21 (17-127)	60.00 ± 12.17 (12-139)
	**Mean time to return (animals hit >1x)**	28.63 ± 11.09 (5-102)	71.50 ± 7.50 (64-79)	73.57 ± 7.23 (52-99)	65.40 ± 17.43 (36-132)	87.67 ± 24.88 (27-254)	52.42 ± 7.19 (23-90)	58.80 ± 9.93 (17-106)	57.00 ± 11.44 (23-106)
	**Mean time to cessation (all)**	NA	110.33 ± 26.96 (79-164)	166.94 ± 21.07 (79-425)	Absent	121.00 ± 26.43 (42-275)	85.56 ± 16.07 (33-194)	120.53 ± 14.77 (46-273)	95.67 ± 12.44 (28-206)
	**Mean time to cessation (animals hit >1x)**	NA	NA	195.67 ± 50.38 (79-425)	NA	135.00 ± 31.24 (60-275)	136.33 ± 29.46 (97-194)	112.56 ± 14.37 (61-184)	92.88 ± 15.32 (28-177)
D&E	**Mean time to return (all)**	53.00 (n=1)	Absent	53.00 (n=1)	Absent	34.00 ± 3.00 (31-37)	24.50 ± 6.54 (10-37)	44.00 (n=1)	20.50 ± 8.50 (12-29)
	**Mean time to return (animals hit >1x)**	53.00 (n=1)	Absent	Absent	Absent	Absent	17.00 (n=1)	Absent	Absent
	**Mean time to cessation (all)**	NA	Absent	59.00 (n=1)	Absent	52.00 ± 12.00 (40-64)	33.00 ± 8.03 (9-42)	87.00 (n=1)	35.50 ± 1.50 (34-37)
	**Mean time to cessation (animals hit >1x)**	NA	Absent	Absent	Absent	Absent	39.00 (n=1)	Absent	Absent
